# Gene-Edited Human-Induced Pluripotent Stem Cell Lines to Elucidate *DAND5* Function throughout Cardiac Differentiation

**DOI:** 10.3390/cells12040520

**Published:** 2023-02-05

**Authors:** José M. Inácio, Mafalda M. Nunes, Micael Almeida, Fernando Cristo, Rui Anjos, José A. Belo

**Affiliations:** 1 Stem Cells and Development Laboratory, iNOVA4Health, NOVA Medical School/Faculdade de Ciências Médicas, Universidade NOVA de Lisboa, 1150-082 Lisboa, Portugal; 2 Hospital de Santa Cruz, Centro Hospitalar Lisboa Ocidental, 1449-005 Lisboa, Portugal

**Keywords:** DAND5, cardiomyocyte proliferation, congenital heart disease, disease modelling

## Abstract

(1) Background: The contribution of gene-specific variants for congenital heart disease, one of the most common congenital disabilities, is still far from our complete understanding. Here, we applied a disease model using human-induced pluripotent stem cells (hiPSCs) to evaluate the function of DAND5 on human cardiomyocyte (CM) differentiation and proliferation. (2) Methods: Taking advantage of our *DAND5* patient-derived iPSC line, we used CRISPR-Cas9 gene-editing to generate a set of isogenic hiPSCs (*DAND5*-corrected and *DAND5* full-mutant). The hiPSCs were differentiated into CMs, and RT-qPCR and immunofluorescence profiled the expression of cardiac markers. Cardiomyocyte proliferation was analysed by flow cytometry. Furthermore, we used a multi-electrode array (MEA) to study the functional electrophysiology of DAND5 hiPSC-CMs. (3) Results: The results indicated that hiPSC-CM proliferation is affected by DAND5 levels. Cardiomyocytes derived from a *DAND5* full-mutant hiPSC line are more proliferative when compared with gene-corrected hiPSC-CMs. Moreover, parallel cardiac differentiations showed a differential cardiac gene expression profile, with upregulated cardiac progenitor markers in DAND5-KO hiPSC-CMs. Microelectrode array (MEA) measurements demonstrated that DAND5-KO hiPSC-CMs showed prolonged field potential duration and increased spontaneous beating rates. In addition, conduction velocity is reduced in the monolayers of hiPSC-CMs with full-mutant genotype. (4) Conclusions: The absence of DAND5 sustains the proliferation of hiPSC-CMs, which alters their electrophysiological maturation properties. These results using DAND5 hiPSC-CMs consolidate the findings of the in vitro and in vivo mouse models, now in a translational perspective. Altogether, the data will help elucidate the molecular mechanism underlying this human heart disease and potentiates new therapies for treating adult CHD.

## 1. Introduction

Human-induced pluripotent stem cells (hiPSCs) from a patient basis are considered a powerful resource for investigating human diseases [1]. Heart disease is a leading cause of premature death or lifelong disabilities in our days [2]. Either adult heart dysfunction caused by coronary vascular disease, arterial hypertension, or congenital heart disease (CHD) contributes to this worldwide health threat [3,4]. 

Heart formation is a complex molecular developmental process, comprising a confluence of multiple pathways and thoroughly sensitive to alterations in key gene dosage [5,6,7,8,9]. Indeed, in the past decade, a multitude of next-generation sequencing studies have been exploring the relationship between genes, gene variants, the expression of these genes and the potential causal link to clinical outcomes when deregulated [7,10,11,12,13]. Overall, the genetic architecture of CHD is being disclosed [12]. But these include the key genes and pathways defined as crucial for the formation and function of the heart, like the TGF-β/Nodal and the WNT/β-catenin signalling pathways [12,14]. However, the casualty of specific gene variants of these pathways in heart disease is still beyond elucidation [15,16].

DAND5 is an extracellular protein that regulates TGF-β/Nodal signalling [17]. This Cerberus/DAN antagonist prevents agonist-receptor interaction by direct binding to the ligand NODAL and/or to NODAL (co)receptors suppressing subsequent signalling activation. Moreover, DAND5 is among the multivalent Cerberus/Dan family members that are also able to bind and inhibit BMP and WNT ligands [17]. In mice, *Dand5* loss-of-function leads to an increased mitotic index of the cardiomyocytes at the compact myocardium, thickening the walls of ventricles causing cardiac dysfunction [18,19,20]. Moreover, DAND5 was assigned as a molecule involved in cardiomyocyte proliferation/differentiation. Reduced DAND5 levels lead to prolonged TGF-β/Nodal and Wnt/β-catenin signalling during mouse embryonic stem cell cardiomyocyte differentiation, increasing cardiovascular progenitors’ number and extending their progenitor state [21].

Regardless, in a genetic screening conducted to understand the role of DAND5 in the aetiology of congenital heart disease, a *DAND5* c.455G/A non-synonymous variant was identified in patients displaying an array of CHD [22]. This non-synonymous variant is localised in the functional domain of the protein (p.R152H), significantly reducing the regular NODAL-inhibitory activity of DAND5 [22].

The differentiation of hiPSCs towards cardiac lineages has become a routine but robust procedure in many laboratories worldwide [23,24,25]. Combined with CRISPR-Cas9 gene-editing technology, it is possible to generate a panoply of isogenically matched sets of either patient-derived or healthy and gene-edited cell lines for disease modelling [26]. Here, we made use of our available set of patient-derived hiPSCs lines, NMSUNLi001-A (DAND5-Var) [27] and NMSUNLi00 3 (DAND5-C) [28], bearing the *DAND5* c.455G/A variant and the isogenic repaired control to study DAND5 activity during cardiomyocyte differentiation, respectively. Our first results indicated that *DAND5*-Var hiPSC line had limitations to efficiently study the impact of DAND5 on human cardiomyocyte proliferation, mainly at latter stages of differentiation. We then successfully generated a *DAND5* full-mutant hiPSC line using CRISPR/Cas9 genome-editing technology. By testing the proliferation, gene expression profile and functional electrophysiology, we demonstrate that *DAND5* inactivation promotes the generation of more proliferative but electrophysiologically competent iPSCs-derived cardiomyocytes. In conclusion, these findings may be used to explore new therapeutic compounds for treating adult CHD.

## 2. Materials and Methods

### 2.1. Human Induced Pluripotent Stem Cell (hiPSC) Maintenance and Gene-Editing

The hiPSC lines were cultured as described previously [29]. Briefly, cells were grown on six-well plates coated with hESC-Qualified Geltrex (Thermo Fisher Scientific, Waltham, MA, USA) in Essential 8 Medium (Thermo Fisher Scientific, Waltham, MA, USA) at 37 °C in a humidified atmosphere (95% air, 5% CO_2_). The medium was daily renewed, and cells were passaged using TrypLE Select (Thermo Fisher Scientific, Waltham, MA, USA) upon reaching 85% confluence. Three isogenic hiPSC lines were used in the experiments: The NMSUNLi001-A (DAND5-Var) which carries a *DAND5* heterozygous c.455G>A non-synonymous variant in the functional domain of DAND5 protein (p.R152H) [27]; the NMSUNLi00 3 (DAND5-C) which is an isogenic hiPSC line with homozygous correction of the c.455G>A alteration in the *DAND5* gene [28]; and the *DAND5*-full knockout (DAND5-KO) hiPSC line (generated here). Using CRISPR/Cas9, a stop codon triad into DAND5 signal peptide region was introduced by homology-directed repair (HDR) as carried out previously for the generation of the isogenic-corrected NMSUNLi003 cell line [28]. Thus, using CRISPR design web tool in crispr.mit.edu, a specific sgRNA was designed and cloned into pCAG-SpCas9-GFP-U6-gRNA plasmid (Addgene #79144). Then, a total of 1.0 × 10^6^ cells were co-nucleofected with 2 µg of the resulting plasmid, and 1 µL of 100 µM ssODN using Neon transfection system (Thermo Fisher Scientific, Waltham, MA, USA), following the manufacturer’s instructions, and cultured in StemFlex Medium (Thermo Fisher Scientific, Waltham, MA, USA) supplemented with RevitaCell Supplement at a 1X final concentration (Thermo Fisher Scientific, Waltham, MA, USA), at 37 °C in a humidified atmosphere (95% air, 5% CO_2_). After additional 2 days, the cells were sorted by FACS and plated at single-cell density into 96-well plates. Clones were screened by sequencing of amplicons spanning the target site of *DAND5* gene exon 1. The positive iPSC clones were expanded and fully characterised as described in [27,28,29]. Karyotype analysis was performed using GTG high-resolution chromosome banding technique at 400–500 band resolution, and 30 metaphases were analysed (service performed by GenoMed, Diagnósticos de Medicina Molecular, SA, Lisboa, Portugal).

### 2.2. Differentiation of hiPSCs into Human Cardiomyocytes

hiPSC-CMs were generated using a robust 2D monolayer differentiation protocol described elsewhere [30], with the adjustments described in [31]. Briefly, ~2 × 10^5^ undifferentiated cells were dissociated and re-plated into 12-well plates coated with hESC-Qualified Geltrex (Thermo Fisher Scientific, Waltham, MA, USA). Cells were cultured in Essential 8 Medium (Thermo Fisher Scientific, Waltham, MA, USA) to 80% cell confluence, and then treated for 24 h with 12 μM CHIR99021, 100 ng/mL Activin A and 270 μM of Ascorbic Acid in RPMI supplemented with B27 serum without Insulin (Thermo Fisher Scientific, Waltham, MA, USA), to induce CM differentiation. On day 1, cells were placed in RPMI + B27 serum without insulin, supplemented with 270 μM of ascorbic acid and 5 μM IWP4. On day 3, the medium was replaced by RPMI + B27 serum without insulin supplemented with 5 μM IWP4. On day 6, cells were removed from treatment and placed on RPMI + B27 without insulin. Finally, from day 7 of differentiation onward, cells were placed in RPMI + B27 serum with insulin, renewed every two-three days until further analysis. Spontaneous beating could be observed from day 8 to 10, consistent with cardiac cell differentiation.

### 2.3. Flow Cytometry

To analyse cardiomyocyte proliferation, newly synthesised DNA was labelled by incubating differentiated cells with EdU for 17 h. EdU detection was performed using Click-iT EdU Alexa Fluor 488 Imaging Kit (Thermo Fisher Scientific, Waltham, MA, USA) according to the manufacturer’s instructions and then incubated with antibodies for cTNT2 at 4 °C for 30 min. Relative fluorescence intensity of cells was detected by Becton Dickinson FACSCanto II (BD Biosciences, Franklin Lakes, NJ, USA), and a minimum of 30,000 events were acquired for each condition. Analysis of the data was performed using FlowJo v10 software (BD Biosciences, Franklin Lakes, NJ, USA).

### 2.4. Quantitative Real-Time PCR (RT-qPCR)

Total RNA isolation from undifferentiated hiPSCs and hiPSC-CMs, at several days of differentiation, was done using TRI Reagent^®^ (Sigma, St. Louis, MI, USA) and the Direct-zolTM RNA MiniPrep Kit (Zymo Research, Irvine, CA, USA) according to the manufacturer’s instructions. The quantity and quality of RNA samples were assessed using a spectrophotometer (Nanodrop 2000, Thermo Fisher Scientific, Waltham, MA, USA). Only samples with 260/280 nm and 260/230 nm ratios equal or superior to 2.0 were considered. cDNA strands were synthesised through reverse transcription reaction using RevertAid Reverse Transcriptase, Oligo (dT) primers, RiboLock RNase Inhibitor and dNTP (Thermo Fisher Scientific, Waltham, MA, USA). Amplification and fluorescent quantification were obtained on an ABI QuantStudio 5 Real-Time PCR System (Thermo Fisher Scientific, Waltham, MA, USA), using a SensiFAST SYBR Lo-ROX mix (BIOLINE, London, UK), and primers listed in Table S1. RT-qPCR reactions were performed in triplicate. Relative quantification of expression was performed using the ddCt method [32], normalised to *GAPDH* and *β-ACTIN*.

### 2.5. Fluorescent Immunocytochemistry

Undifferentiated or differentiated hiPSCs were fixed in 4% paraformaldehyde, incubated with primary antibodies (diluted in 1x PBS, 1% BSA, 0.05% sodium azide solution) overnight at 4 °C, listed in Table S2, followed by appropriated secondary antibody incubation, overnight at 4 °C. Nuclei were stained with DAPI at room temperature and mounted on glass slides with VECTASHIELD^®^ Antifade Mounting Medium. Cell images were acquired with Zeiss Axio Imager Z2 microscope or Zeiss LSM710 confocal microscope (Zeiss, Oberkochen, Germany). Images were taken in sequential mode and posteriorly adjusted in ImageJ.

### 2.6. Electron Microscopy

All reagents and materials were purchased from electron microscopy sciences unless otherwise stated. Glass coverslips containing cardiomyocyte monolayers were fixed in 2% paraformaldehyde, 2% glutaraldehyde in 0.1 M phosphate buffer (PB) at pH 7.4 overnight at 4 °C. After washing with PB, specimens were post-fixed for 1 h on ice using 1% osmium tetroxide and 1.5% potassium ferrocyanide in distilled water, and then incubated with 1% tannic acid in distilled water for 20 min at room temperature. Specimens were subsequently dehydrated with a series of increasing ethanol concentrations (35%, 45%, 50%, 70%, 90%, 2× 100%) before infiltrating and embedding upon Epon resin stubs (EMbed 812). After polymerising at 65 °C overnight, glass was removed by immersion in liquid nitrogen, and cell monolayers were sectioned on face at 75 nm thickness using a UC7 ultramicrotome (Leica, Wetzlar, Germany) and a diamond knife (Diatome, Nidau, Switzerland), and sections collected on formvar-coated copper mesh grids. Sections on grids were then sequentially post-stained with 2% uranyl acetate in 70% methanol followed by Reynold’s lead citrate and imaged using a Hitachi H-7650 TEM equipped with an AMT XR41M digital camera.

### 2.7. Microelectrode Array Measurements

The in vitro electrophysiological recordings were performed on a prime high-density microelectrode array (Prime HD-MEA) from 3Brain AG, Pfaffikon, Switzerland. This Prime HD-MEA chip integrates 4096 recording electrodes on a small and compact area of 2.67 mm × 2.67 mm, centred on a flat area of ~3 mm × 3 mm. The hiPSC-CMs were plated on coated MEA chips, with laminin (0.1 mg/mL), poly-d-lysine (0.1 mg/mL) and Geltrex (15 mg/mL). The MEAs were placed in an incubator with a temperature of 37 °C and 5% CO_2_ and were given six days to ensure that the hiPSC-CMs were well attached to the MEA. Recordings were performed on days 21 and 51 of the cardiac differentiation and acquired at 10 Hz for 2 min. During recordings, the temperature was kept at 37 °C, and data were recorded and analysed using BrainWave 4 Software (Version 4.0).

### 2.8. Electrophysiological Parameter Analysis

To analyse the electrophysiological parameter, the analysis tools Spike Detection, Spike Sorting and Local Field Potential (LFP) in the BrainWave 4 Software (Version 4.0) were used. For the spike detection, a hard threshold of −300 µVolt and a minimum refractory period of 1 ms was used; for spike sorting, pre- and post-event of 3 ms was used; and finally, for the LFP detection, a lower threshold of −300 µVolt and a max event duration of 600 ms were set. Additionally, the cardiac extractor and the mean waveform were used for python analyses. With these analyses, we were able to extract several parameters, which were the case of field potential duration, spontaneous beating rate, mean waveforms of field potential and conduction velocity. Conduction velocities were calculated by dividing the distance between the two electrodes by propagation time. All data sets are represented as median ± SD compiling more than 3000 chosen electrodes from four independent biological experiments.

### 2.9. Statistical Analysis

The statistical analyses were performed using GraphPad Prism version 8.0 for Mac (GraphPad Software). Statistical significance was determined by unpaired Students t-test to compare two groups. Differences were considered statistically significant when * *p* < 0.05, ** *p* < 0.01, *** *p* < 0.001, **** *p* < 0.0001. All statistical parameters are reported in the respective figures and figure legends.

## 3. Results

### 3.1. Disease Model System for DAND5 Studies

Based on distinct mouse model studies, it becomes evident that *DAND5* is associated with gene networks controlling differentiation and cell cycle of cardiomyocytes at early developmental stages [18,21]. The inactivation of *Dand5* in cardiac populations increases the proliferative capacity of cardiomyocytes during mouse cardiac development [18] and mouse ES-CM differentiation [21].

**Figure 1:**
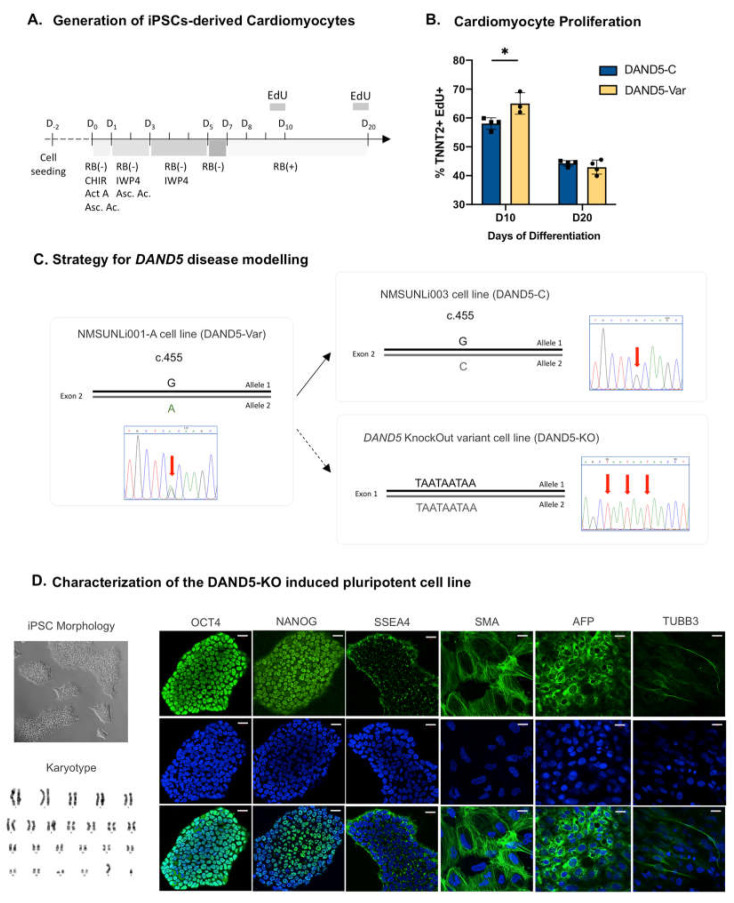
*DAND5* disease modelling setting. (**A**) Schematic representation of the differentiation process, depicting the small molecules added and EdU incubation. (**B**) Analysis of TNNT2-positive cell proliferation in DAND5-Var (yellow) vs. DAND5-C (blue) iPSC-CM by labelling newly synthesised DNA using EdU incubation for 17 h. The relative fluorescence intensity of cells was analysed by flow cytometry at day 10 (D10) and 20 (D20) of differentiation. (**C**) Strategy used for the generation of the different hiPSCs using CRISPR/Cas9 technology. (**D**) Characterisation of DAND5-KO cell line: Left panel—(top) bright-field image of the DAND5-KO cell colonies morphology; (bottom) representative metaphase showing normal 46 chromosomes (XY) karyotype. Middle panels—immunodetection of undifferentiated markers *OCT4*, *NANOG* and *SSEA4* of DAND5-KO hiPSCs (scale bar: 40 μm). Right panels—immunofluorescence for endodermal marker α-fetoprotein (AFP), ectodermal marker βIII-tubulin (TUBB3) and mesodermal marker α-smooth muscle actin (SMA) showing DAND5-KO hiPSCs trilineage differentiation potentiality (scale bar: 20 μm). Nuclei were stained with DAPI. All the results represent the mean ± SD of three independent biological experiments. Unpaired Student’s t-test was applied to compare the differences between DAND5-C and DAND5-Var groups on each day of differentiation. Statistically significant results were considered when * *p* < 0.05.

To address whether DAND5 could undergo a cell cycle change in hiPSC-CMs, we started by studying the proliferation rate of cardiomyocytes derived from our two cell lines NMSUNLi001-A and NMSUNLi003, a *DAND5* patient-derived and its isogenic-corrected cell lines, respectively (Figure 1A). The NMSUNLi001-A cell line (DAND5-Var) carries a *DAND5* heterozygous c.455G>A non-synonymous variant in the functional domain of DAND5 protein (p.R152H) [27]; the NMSUNLi003 is an isogenic hiPSC line, *DAND5*-Corrected (DAND5-C) with homozygous correction of the c.455G>A alteration in the *DAND5* gene, using CRISPR/Cas9 technology [28]. It is known that the proliferation rate decreases during cardiomyocyte differentiation but with a continuous increase of TNNT2^+^ cells during the first weeks of differentiation [33,34]. Here, our cell proliferation assay showed that there is a significant increase in the proliferation of the DAND5-Var CMs at day 10 of differentiation (Figure 1B). However, at day 20 of differentiation, this difference lessens (Figure 1B). One most likely interpretation for this result is the functional efficiency level of DAND5 protein in the DAND5-Var cell line when compared with the isogenic-corrected cell line DAND5-C. DAND5-Var cell line carries a *DAND5* heterozygous c.455G>A non-synonymous variant in the functional domain of DAND5 protein (p.R152H), which reduces the normal inhibitory activity of this antagonist [22]. As such, the reduction in proliferation at initial differentiation stages seems not to strike a significant difference between these cell lines at later stages of differentiation. As the resulting DAND5-Var protein is just affected in its efficiency to inhibit NODAL activity, maybe this is not enough to fully uncover experimentally the required function of DAND5 in human cardiomyocyte proliferation, mainly at latter stages when proliferation is already normally lower (Figure 1B).

In order to better address this question, it becomes essential to generate an isogenic *DAND5*-full knockout (DAND5-KO) hiPSC line. Using CRISPR/Cas9-mediated genome editing, we were able to introduce a stop codon triad into DAND5 signal peptide region by homology-directed repair (HDR) and confirmed by DNA Sanger sequencing analysis (Figure 1C). This technology has been used previously for the generation of the isogenic-corrected NMSUNLi003 cell line [28]. The DAND5-KO hiPSCs showed typical human embryonic stem cell morphology with a high nucleus/cytoplasm ratio, karyotypically normal and expressed the undifferentiated cell markers *NANOG*, *OCT4* and *SSEA4* (Figure 1D). In vitro embryoid body-based differentiation was used to assign differentiation capacity into the three germ layers. Immunofluorescence analysis revealed a positive signal for the endodermal marker α-fetoprotein (AFP), the mesodermal marker smooth muscle actin (SMA) and the ectodermal marker βIII-tubulin (TUBB3), indicating that DAND5-KO hiPSCs spontaneously differentiate to the endoderm, mesoderm and ectoderm lineages, respectively (Figure 1D). These results indicate that DAND5-KO hiPSC line has been successfully generated and represents a new tool to study in vitro the role of DAND5 in human cardiomyogenesis.

### 3.2. Proliferation of hiPSC-CMs Is Influenced by DAND5

**Figure 2:**
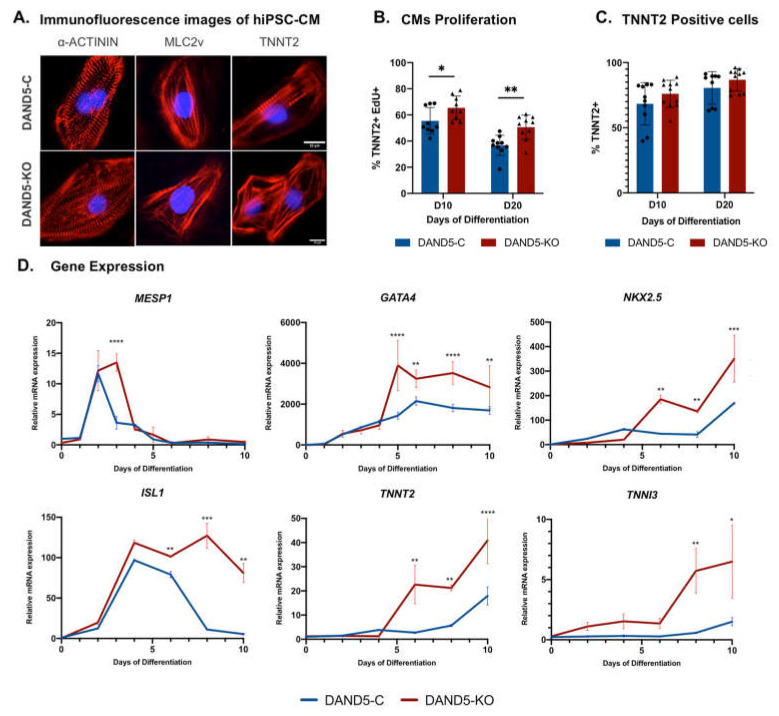
DAND5 depletion leads to higher rates of cardiomyocyte proliferation. (**A**) Representative immunofluorescence images of hiPSC-CM from DAND5-C and DAND5-KO cell lines at day 15 for the CM-specific markers: sarcomeric α-ACTININ, MLC2v, TNNT2 (red). Nuclei were counterstained with DAPI (blue). (**B**) Analysis of TNNT2-positive cell proliferation by labelling newly synthesised DNA using EdU incubation for 17 h using flow cytometry. (**C**) Percentage of TNNT2-positive cells analysed by flow cytometry at day 10 (D10) and 20 (D20) of differentiation. (**D**) Relative mRNA expression of *MESP1*, *GATA4*, *NKX2.5*, *ISL1*, *TNNT2*, *TNNI3* genes in DAND5-C and DAND5-KO differentiated cells. All the results represent the mean ± SD of, at least, three independent biological experiments. Unpaired Student’s t-test was applied to compare the differences between the DAND5-C and DAND5-KO groups on each day of differentiation. Statistically significant results were considered when * *p* < 0.05, ** *p* < 0.01, *** *p* < 0.001 and **** *p* < 0.0001.

To test how DAND5 affects the differentiation and cell cycle of human cardiomyocytes, we differentiate DAND5-C and DAND5-KO hiPSC lines into cardiomyocytes using the monolayer differentiation protocol used before (Figure 1A) [31]. Spontaneously contracting foci were observed around 8 to 10 days of cardiac differentiation, and the CMs generated from both iPSC cell lines exhibited positive staining of structural cardiac-specific markers α-ACTININ, MLC2v and TNNT2 (Figure 2A). We thus further assessed the influence of DAND5 on human cardiomyocyte proliferation using the Click-iT EdU assay. A significant difference in the TNNT2^+^EdU^+^ population was observed between the DAND5-C and DAND5-KO hiPSC lines (Figure 2B). Moreover, this difference in proliferation rate is sustained at day 20 of differentiation (Figure 2B), in contrast to what was observed between DAND5-Var and DAND5-C lines (Figure 1B). Additionally, DAND5-KO hiPSC line showed evidence of a higher percentage of TNNT2^+^ cells over time (Figure 2C). Figure 2D shows the expression of relevant cardiac marker genes during the first 10 days of differentiation obtained by RT-qPCR. Most of the genes tested, *MESP1*, a well-known cardiogenic mesoderm marker, the cardiac-specific progenitors *GATA4*, *NKX2.5* and *ISL1*, and two structural sarcomeric-related genes, *TNNT2* and *TNNI3*, showed a significantly increased expression in DAND5-KO hiPSC-CMs. Altogether, the results revealed that *DAND5* ablation promotes the generation of more proliferative iPSCs-derived cardiomyocytes.

### 3.3. Structural and Molecular Analysis of DAND5-KO hiPSC-CMs

As previously mentioned, a former study performed on mouse embryonic stem cells, has demonstrated that the suppression of DAND5 leads to an increase in proliferation of cardiomyocytes and drives to a distinct transcriptional profiling sequencing during the first stages of life [21]. In line with this study, we performed an extended structural and molecular analysis of DAND5-KO hiPSC-CMs.

**Figure 3:**
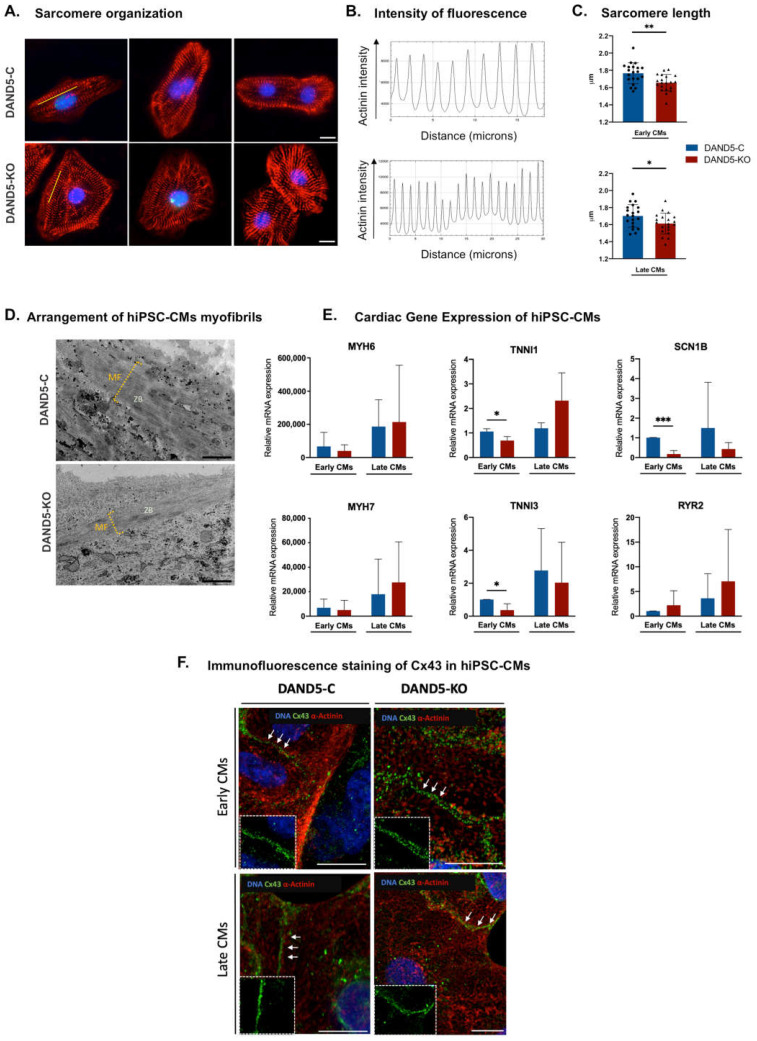
Structural and molecular analyses of DAND5 hiPSC-CM. (**A**) Representative immunofluorescence images of hiPSC-CM from DAND5-C and DAND5-KO cell lines at day 15 for sarcomeric α-ACTININ (red). Scale bar: 10 µm. Nuclei were counterstained with DAPI (blue). (**B**) The sarcomere was measured by tracing a line (yellow) across sarcomeres using Fiji software and extracting fluorescence intensity. (**C**) Distribution of sarcomeres sizes, at early (day 21) and late (day 51) stages. *n* = 20 cells for each cell line. (**D**) Representative transmission electron microscopy (TEM) images showing arrangement of sarcomeres in both hiPSC-CMs lines, scale bar: 500 nm, MF—Myofibrils; ZB—Z-bodies. (**E**) Relative mRNA expression of, *MYH6*, *MYH7*, *TNNI1*, *TNNI3, SCN1B* and *RYR2* genes in DAND5-C and DAND5-KO differentiated cells, at early (day 21) and late (day 51) stages, from at least three independent biological experiments. Only *MYH6* and *MYH7* were normalised for hiPSC, the remaining genes were normalised to the respective DAND5-C of each biological replicate. (**F**) Immunofluorescence of CX43 (green), α-ACTININ (red) and nuclei are stained with DAPI (blue) in DAND5-C and DAND5-KO differentiated cells at early (day 21) and late (40 days) stages. Insets are magnifications of the areas depicted by the white arrows to highlight CX43 distribution. Scale bar: 10 µm. Unpaired Student’s t-test was applied to compare the differences between DAND5-C and DAND5-KO groups. Statistically significant results were considered when * *p* < 0.05, ** *p* < 0.01 and *** *p* < 0.001.

First to study whether the extended proliferation window of DAND5-KO hiPSC-CMs influenced or not the cardiomyocyte structure, we analysed the organisation of the sarcomeres during the differentiation process by antibody labelling for sarcomeric α-ACTININ. We observed that DAND5-C hiPSC-CMs presented more organised and aligned sarcomeres compared to DAND5-KO hiPSC-CMs (Figure 3A). By measuring sarcomere length in confocal microscopy images, we noticed that DAND5-C hiPSC-CMs displayed the longest average sarcomere length, while DAND5-KO hiPSC-CMs presented shorter sarcomeres and the greatest proportion of disorganised sarcomeres (Figure 3A–C). Moreover, ultrastructural imaging showed that DAND5-C hiPSC-CMs contain well-organised and thicker myofibrils, while the DAND5-KO hiPSC-CM’s myofibrils were thinner and lesser arranged (Figure 3D). These indicate that the DAND5-C hiPSC-CMs have a higher level of structural maturation, which facilitates force generation [35]. Next, we examined the expression of *TNNI1*, *TNNI3*, *MYH6*, *MYH7*, *SCN1B* and *RYR2* at the transcriptional level using RT-qPCR (Figure 3E). Throughout the process of hiPSC-CMs maturation, it occurs an isoform transition of sarcomeric genes, such as in the case of the cardiac myosin heavy chains, that switch from the alpha (*MYH6*) expression to the beta isoform (*MYH7*) expression, and the troponin I, that switches from the slow skeletal (encoded by *TNNI1*) to the cardiac isoform (encoded by *TNNI3*) [36,37,38,39,40]. First, we observed that in both hiPSC-CMs lines *MYH7*, *MYH6, TNNI1* and *TNNI3* expression levels augment over the time course (Figure 3D). Nevertheless, there is a clear switch in the troponin I isoforms, trending towards a *TNNI3/TNNI1* ratio increase in the DAND5-C hiPSC-CMs (Early/Late CMs: 0.95/ 2.33) when compared with DAND5-KO hiPSC-CMs (Early/Late CMs: 0.54/ 0.88). The RT-qPCR analyses were also used for investigating the mRNA expression levels of a sodium voltage-gated channel, *SCN1B* and a calcium receptor, the Ryanodine receptor 2, *RYR2* [41,42]. Regarding *SCN1B*, we observed a clear increase in DAND5-C hiPSC-CMs and a slighter increase in DAND5-KO, over the differentiation (Figure 3E). Concerning *RYR2* expression levels, we observed that, over the time course, the DAND5-KO exhibits a slightly increased expression of *RYR2* when compared with DAND5-C hiPSC-CMs (Figure 3E).

In addition, recent studies have demonstrated the importance of a cardiac gap junction, the connexin 43 (Cx43) responsible for a proper electrical propagation between cells [43]. By performing an immunofluorescence analysis using antibodies against this connexin, we observed that both hiPSC-CMs lines expressed Cx43 (Figure 3F). However, no apparent difference in Cx43 accumulation or localisation were noticed between hiPSC-CM lines (Figure 3F).

These results suggest that upon genetic inactivation of *DAND5*, the produced hiPSC-CMs demonstrated minor sarcomeric maturity, presenting less organised and significantly smaller sarcomeres. When considering gene expression, the results are not so much distinctive, but there is a clear increase in *SCN1B* expression levels in DAND5-C and *TNNI3/TNNI1* ratio relative to DAND5-KO phenotype. Consequently, it is not surprising that the DAND5-C hiPSC-CMs and DAND5-KO hiPSC-CMs could display differential electrophysiological properties.

### 3.4. DAND5 Level Affects the Electrophysiological Maturation Process of hiPSC-CMs

**Figure 4:**
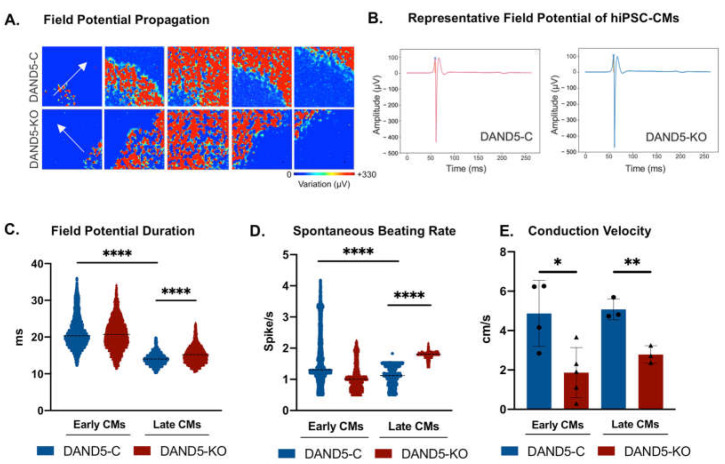
Electrophysiological analysis of DAND5 hiPSC-CMs using MEA. (**A**) Snapshots of MEA recordings show spontaneous FP, which propagates across a monolayer of DAND5-C (top panel) and DAND5-KO (bottom panel) cells. The white arrow denotes the direction of FP propagation. FPs were recorded with a high-pass filter of 50 Hz for 2 min. (**B**) Mean waveform of FP of the DAND5-C (pink) and DAND5-KO (blue) lines. The mean waveform was obtained by selecting active electrodes displaying a variation higher than 330 μVolts using BrainWave 4 Software and in house Python script. (**C**) Field potential duration. (**D**) Spontaneous beating rate. (**E**) Conduction velocity. Data represented the scatter plot of all individual values of four independent biological experiments. Unpaired Student’s t-test was applied to compare the differences between the DAND5-C and DAND5-KO groups on each day of differentiation. Statistically significant results were considered when * *p* < 0.05, ** *p* < 0.01, and **** *p* < 0.0001.

We further evaluated the electrophysiological properties of DAND5 hiPSC-CMs using the high-density microelectrode array (HD-MEA) to record extracellular field potential (FP), whose waveforms reliably correlate with the cardiac action potential and closely resemble electrocardiogram recordings [44]. Figure 4A shows that the hiPSC-CMs from both lines demonstrated spontaneous FP signal that propagated throughout the cell monolayer suggesting the formation of a functional syncytium. Representative FP from DAND5-C and DAND5-KO hiPSC-CMs are shown in Figure 4B, visibly associated with mean waveforms of ventricular-like CMs [45]. Statistical analysis showed that late DAND5-KO hiPSC-CMs presented significantly prolonged field potential duration (FPD), the time from the depolarisation to repolarisation, when compared with DAND5-C hiPSC-CMs (Figure 4C), which is indicative of a more foetal-like phenotype [46]. Moreover, we noticed that the spontaneous beating rate displayed by hiPSC-CMs from the DAND5-C decreased throughout differentiation (Figure 4D). Unexpectedly, DAND5-KO hiPSC-CMs showed an increasing trend in the spontaneous beating frequency throughout the time course (Figure 4D). In addition, we assessed the conduction velocity of the beating hiPSC-CM syncytium (Figure 4E), which indicates how the electrical signal propagates in the cardiac tissue. The measurements demonstrated that the electrical impulse propagated with faster conduction velocities in DAND5-C hiPSC-CM monolayers throughout differentiation (Figure 4E). Interestingly, this faster conduction velocity in DAND5-C is compatible with the high expression levels of *SCN1B* in this cell line compared with the DAND5-KO (Figure 3E). Taken together, the electrophysiological parameters showed that the inactivation of *DAND5* delayed the progression of cardiac maturity, which follows all previous results.

## 4. Discussion

In this work, we provided a hiPSCs model to evaluate the activity of DAND5 during human-based cardiogenesis, contributing to the understanding of congenital heart disease mechanisms. DAND5, which is expressed in the developing heart, is a well-known antagonist of TGF-β/Nodal signalling but is also implicated in BMP and WNT signalling [17,18,47], three signalling pathways that actively participate in heart development [48,49,50,51]. Mutations in *DAND5*, such as the heterozygous c.455G>A missense mutation, which decreases DAND5 activity, and the homozygous truncating c.396_397dupCT variant predicted to produce a frameshift protein, have been strongly associated with clinically diagnosed heart malformations [14,22]. In mouse models, it was noticed that, among other cardiac defects, *Dand5* loss-of-function promotes the proliferation of cardiomyocytes [18,21]. The hearts of *Dand5* knockout mice display ventricular hypertrophy with increased ventricular wall thickness, which causes severe cardiac dysfunction [18]. Moreover, using mouse embryonic stem cells (mESCs), it was shown that *Dand5* loss-of-function dramatically increased the number of FLK-1+/PDGFR-α+ cardiac progenitor cells during mESCs cardiac differentiation [21]. In addition, the knockout of *Dand5* seems to activate the transcription of several cell-cycle regulators, prompting cardiomyocyte proliferation [18,21]. Here, our cell proliferation assays demonstrate for the first time in a human model that reduced DAND5 levels significantly enhanced cardiomyocyte proliferation. Cardiomyocyte differentiation is a complex dynamic mechanism that depends on the tight control of gene expression patterns during the process of cardiac mesoderm formation, cardiac progenitor phase, and successfully, functional cardiomyocyte establishment [52,53,54,55,56]. In fact, our hiPSC model indicates a correlation between DAND5 activity and cardiomyocyte proliferation. The percentage of TNNT2^+^/EdU^+^ cells is, and remains, higher in the differentiation of the DAND5-KO cell line when compared with the DAND5-Var cell line, in which DAND5 activity is reduced but not absent [22]. In addition, the cumulative increase in the number of CMs every day during the 20 days of differentiation will result in a substantial increase in the total number of iPSC-CMs derived from the DAND5-KO line, when compared with those generated from the DAND5-C line. Moreover, gene expression profiling of DAND5 hiPSC lines exhibits a differential cardiac gene expression profile of key markers. We found that *MESP1*, *ISL1*, *NKX2.5* and *GATA4* were upregulated during the differentiation of the DAND5-KO cell line compared to the control line, DAND5-C. A recent study showed that MESP1+ human embryonic stem-derived cells highly express heart development genes promoting robust cardiomyocyte production [57]. ISl1 identifies a proliferating cardiac population that contributes most cells to the heart [58,59]. Still, it is also involved in cardiomyocyte proliferation together with the Brg1-Baf60c-based SWI/SNF complex [60]. *NKX* genes are a well-known family of genes that regulate ventricular and atrial cell numbers during heart development [61]. More recently, it has been reported that NKX2.5 is required for myocardial regeneration by controlling cardiomyocyte dedifferentiation and proliferation upon cardiac injury [62]. GATA4 is a mutual cofactor of NKX2.5 and has been shown to regulate myocardial proliferation by activating cyclin D2 and CDK4 [63,64,65]. Altogether, the altered molecular profiles in *DAND5* loss-of-function illustrate a more proliferative state of the DAND5-KO hiPSC-CMs, which is clearly concomitant with the higher number of TNNT2^+^ cells obtained in the full-mutant cell line. Moreover, our previous work assigned that DAND5 levels lead to prolonged TGF-β/Nodal and WNT/β-catenin signalling during mouse heart development and mESC-CMs derivation, which not only increases cardiovascular progenitors’ levels but also extends their progenitor state [18,19,21]. Interestingly, it has been shown that the dual knockdown of two other NODAL signalling antagonists, CER1 (CERBERUS-1) and LEFTY1 (left-right determination factor 1) in hPSCs, resulted also in an increase in NKX2.5-GFP^+^ cells [66].

Electrode electrophysiology has been used in several studies to dissect currents and ions in iPSC-CMs [67,68,69,70,71,72]. Summed up, it is accepted that several electrophysiological parameters, such as action potential duration or drug interactions, can be tested in hiPSC-CMs since they present an electrical activity similar to human cardiac myocytes. However, it is important to note that in assessing disease phenotype using iPSC-CMs from genetic backgrounds, caution is advised when drawing assumptions. A recent study, modelling short QT Syndrome highlighted the importance of using isogenic control hiPSC in electrophysiology studies [69]. Here, we used MEA to extract the FP waveform of the isogenic DAND5 hiPSCs lines at different phases of cardiac differentiation. The distinction between ventricular-like, atrial-like, or pacemaker-like cells could be extrapolated from FP waveform shape [73]. Moreover, the FP waveform shape reflects the electrical function of the expressed ion channels, and even slight alterations in their well-regulated ion conductance can significantly influence FP morphology [74]. Per the mean waveforms obtained in this work, most of the hiPSC-CMs detected exhibited the electrical physiology of ventricular-like CMs [45]. Furthermore, we observed statistically significant longer FPD in the late DAND5-KO hiPSC-CMs compared to that in DAND5-C hiPSC-CMs. Nevertheless, both lines decreased their FPD with prolonged culture. Curiously, patch-clamp studies previously reported that the action potential duration (APD) of human foetal CMs is 20–80% longer than for adult CMs [46]. Nevertheless, the numbers obtained here are in the lower dimension of this difference. Correlating these data, the longer FPD values displayed by DAND5-KO hiPSC-CMs suggest more foetal-like behaviour of these cardiomyocytes. Regarding the spontaneous beating rate data, we noticed that the DAND5-C hiPSC-CMs beating frequency decreased throughout differentiation. In contrast, the DAND5-KO line presented an increased beating frequency when compared with the DAND5-C hiPSC at latter cardiac differentiation phases. As expected, the decrease observed in spontaneous beating rate for the DAND5-C line is consistent with the increased expression of the slower β-isoform (MYH7) of myosin heavy chain in DAND5-C hiPSC-CMs. In the foetal stages of the heart, *MYH6* is highly expressed and is characterised by its fast ATPase activity and rapid actin-binding features that support the faster beating in the early stages, and then, with development, expression gradually shifts to express the *MYH7* isoform [75,76,77]. Moreover, the troponin I isoform switching from slow skeletal troponin I (TNNI1) to the cardiac troponin I (TNNI3), which is less sensitive to Ca^2+^, also affects contractility decreasing spontaneous beating rate [78]. On the other hand, ryanodine receptors (RYR) play a critical role in intracellular Ca^2+^ modulation [79]. Augmented contractility rate is preceded by RYR2 gain of function [80], which might account also for the increased beating frequency seen in DAND5-KO hiPSC-CMs. Moreover, we noticed that in the DAND5-C hiPSC-CM monolayers, the electrical impulse propagated with faster conduction velocities than in the DAND5-KO hiPSC-CM syncytium, which is a sign of less maturity of the full-mutant CMs. Indeed, it has been shown that the conduction velocity hiPSC-CMs is rather slower (1.5–20 cm/s) compared to human adult ventricular CMs (60 cm/s) [81,82,83], which display a fast and coordinated propagation of electrical activity in healthy hearts [84]. For that, the cardiomyocytes laid in cardiac tissue are tightly connected with each other through gap junctions [85]. Connexin 43 (Cx43) gap junctions are the most abundant gap junction channels in ventricular CMs. Upon biogenesis, Cx43 molecules traverse along actin filaments and microtubules extending from the trans-Golgi network throughout the cell surface in immature CMs, or become polarised to cell termini during in vivo maturation, forming intercalated discs [86]. Therefore, besides the characteristic pattern of punctuated distribution of Cx43 at gap junctions along the cell–cell adjacent membranes, the signal distribution of this connexin is also observed in the cytoplasm near the nucleus in both DAND5-C and DAND5-KO hiPSC-CMs (Figure 3F). Those Cx43 patterns, resemble what has been observed in other hiPSC-derived CMs [76,87,88]. However, although the Cx43 subcellular localisation detected on both lines denoted the foetal-like properties of these hiPSC-derived CMs, the two DAND5 hiPSC-CM lines evidenced discrete cell–cell electrical coupling, displaying conduction velocities ranging from DAND5-KO ~2.0 cm/s to DAND5-C ~5.0 cm/s [89,90]. Curiously, it has been shown in cardiac microtissues that the localisation of Cx43 gap junctions is important for sarcomere organisation [89]. Concomitantly, the TEM images obtained from both DAND5 hiPSC-CMs suggested that the contractile machinery of these CMs is composed of low-density myofibrils and Z-bodies associated with immature CMs rather than more-mature sarcomeric structures with clear Z-disks such as those found in adult CMs [76,91]. Nevertheless, myofibrils in the DAND5-C hiPSC-CM appear to be more uniform and thicker than those displayed by the DAND5-KO hiPSC-CM, evidencing the different phenotype displayed by these DAND5 hiPSC-CM lines. However, on-face myofibril’s ultrastructure with clearly aligned Z-discs is technically easier to image on well-organised cardiac tissues than that on hiPSC-CMs cultured on monolayers, which could represent a limitation in our study [90,92]. In contrast, the faster conduction velocity recorded in DAND5-C hiPSC-CMs is compatible with the high expression levels of *SCN1B* displayed by this cell line, compared with the DAND5-KO hiPSC-CMs. SCN1B is a subunit of Na_v_-β channels cluster that localises at cell–cell junction sites contributing to the action potential conduction across cardiac tissue [93].

Overall, these electrophysiology analyses suggest that both cell lines have different behaviours regarding FPD and spontaneous beating rate, indicating that DAND5-C hiPSC-CMs seem to have characteristics of more mature CMs, and DAND5-KO hiPSC-CMs characteristics of more proliferative CMs. This is in line with previous studies in mouse models, in which it was demonstrated that suppression of DAND5 leads to an augment in the proliferative state of mESCs-derived CMs and also of CMs in the compact myocardium of embryonic and neonatal mouse hearts [18,21]. Curiously, the different trends at cardiac gene expression level described here, between DAND5-C and DAND5-KO hiPSC-CMs, are quite similar to those resulting from previous global transcriptome analysis between *Dand5* KO and WT mESCs [21]. Nevertheless, surviving mutant animals reached adulthood and are fertile [94]. This evidence suggests that although more proliferative, DAND5-KO CMs must undergo an exquisite maturation process as they are suitable for extrauterine life workload demanding. Moreover, the underlying molecular mechanism might be of greater interest to future regenerative medicine applications.

## 5. Conclusions

Here, we demonstrated for the first time the effect of DAND5 during the cardiac differentiation of human-induced pluripotent stem cells. Together, the results showed that *DAND5* loss-of-function promotes the generation of more proliferative hiPSCs-derived cardiomyocytes although electrophysiologically competent. Moreover, we demonstrated that the hiPSCs-based disease model developed here is a high-throughput strategy to study genotype (patient-variants)–phenotype relationship, in complex and polygenic disorders such as CHD. In conclusion, the findings evidenced in this work will be used to investigate new therapeutic compounds for treating adult CHD.

## Data Availability

The data presented in this study are freely available on request from the corresponding author.

## Supplementary Materials

The following supporting information can be downloaded at: https://www.mdpi.com/article/10.3390/cells12040520/s1, Table S1: Primer List; Table S2: Antibody List.

## References

[1] Brooks I.R., Garrone C.M., Kerins C., Kiar C.S., Syntaka S., Xu J.Z., Spagnoli F.M., Watt F.M. (2022). Functional genomics and the future of iPSCs in disease modeling. Stem Cell Rep..

[2] Virani S.S., Alonso A., Benjamin E.J., Bittencourt M.S., Callaway C.W., Carson A.P., Chamberlain A.M., Chang A.R., Cheng S., Delling F.N. (2020). Heart disease and stroke statistics—2020 update: A report from the American Heart Association. Circulation.

[3] Kwan G.F., Mayosi B.M., Mocumbi A.O., Miranda J.J., Ezzati M., Jain Y., Robles G., Benjamin E.J., Subramanian S.V., Bukhman G. (2016). Endemic Cardiovascular Diseases of the Poorest Billion. Circulation.

[4] Triedman J.K., Newburger J.W. (2016). Trends in Congenital Heart Disease: The Next Decade. Circulation.

[5] Bruneau B.G. (2013). Signaling and transcriptional networks in heart development and regeneration. Cold Spring Harb. Perspect. Biol..

[6] Buckingham M., Meilhac S., Zaffran S. (2005). Building the mammalian heart from two sources of myocardial cells. Nat. Rev. Genet..

[7] Kathiriya I.S., Nora E.P., Bruneau B.G. (2015). Investigating the transcriptional control of cardiovascular development. Circ. Res..

[8] Meilhac S.M., Lescroart F., Blanpain C., Buckingham M.E. (2014). Cardiac cell lineages that form the heart. Cold Spring Harb. Perspect. Med..

[9] Meilhac S.M., Buckingham M.E. (2018). The deployment of cell lineages that form the mammalian heart. Nat. Rev. Cardiol..

[10] Belkadi A., Bolze A., Itan Y., Cobat A., Vincent Q.B., Antipenko A., Shang L., Boisson B., Casanova J.-L., Abel L. (2015). Whole-genome sequencing is more powerful than whole-exome sequencing for detecting exome variants. Proc. Natl. Acad. Sci. USA.

[11] Lahm H., Jia M., Dreßen M., Wirth F., Puluca N., Gilsbach R., Keavney B.D., Cleuziou J., Beck N., Bondareva O. (2021). Congenital heart disease risk loci identified by genome-wide association study in European patients. J. Clin. Investig..

[12] Morton S.U., Quiat D., Seidman J.G., Seidman C.E. (2022). Genomic frontiers in congenital heart disease. Nat. Rev. Cardiol..

[13] Yamada S., Nomura S. (2020). Review of Single-Cell RNA Sequencing in the Heart. Int. J. Mol. Sci..

[14] Bolkier Y., Barel O., Marek-Yagel D., Atias-Varon D., Kagan M., Vardi A., Mishali D., Katz U., Salem Y., Tirosh-Wagner T. (2022). Whole-exome sequencing reveals a monogenic cause in 56% of individuals with laterality disorders and associated congenital heart defects. J. Med. Genet..

[15] Richards S., Aziz N., Bale S., Bick D., Das S., Gastier-Foster J., Grody W.W., Hegde M., Lyon E., Spector E. (2015). Standards and Guidelines for the Interpretation of Sequence Variants: A Joint Consensus Recommendation of the American College of Medical Genetics and Genomics and the Association for Molecular Pathology. Genet. Med. Off. J. Am. Coll. Med. Genet..

[16] Strande N.T., Riggs E.R., Buchanan A.H., Ceyhan-Birsoy O., DiStefano M., Dwight S.S., Goldstein J., Ghosh R., Seifert B.A., Sneddon T.P. (2017). Evaluating the Clinical Validity of Gene-Disease Associations: An Evidence-Based Framework Developed by the Clinical Genome Resource. Am. J. Hum. Genet..

[17] Belo J.A., Bachiller D., Agius E., Kemp C., Borges A.C., Marques S., Piccolo S., De Robertis E.M. (2000). Cerberus-like is a secreted BMP and nodal antagonist not essential for mouse development. Genesis.

[18] Araújo A.C., Marques S., Belo J.A. (2014). Targeted inactivation of Cerberus like-2 leads to left ventricular cardiac hyperplasia and systolic dysfunction in the mouse. PLoS ONE.

[19] Belo J.A., Marques S., Inácio J.M. (2017). The Role of Cerl2 in the Establishment of Left-Right Asymmetries during Axis Formation and Heart Development. J. Cardiovasc. Dev. Dis..

[20] Vinhas M., Araújo A.C., Ribeiro S., Rosário L.B., Belo J.A. (2013). Transthoracic echocardiography reference values in juvenile and adult 129/Sv mice. Cardiovasc. Ultrasound.

[21] Inácio J.M., Lopes J.v.G., Silva A.M., Cristo F., Marques S., Futschik M.E., Belo J.A. (2021). DAND5 inactivation promotes cardiac differentiation in mouse embryonic stem cells. Front. Cell Dev. Biol..

[22] Cristo F., Inácio J.M., de Almeida S., Mendes P., Martins D.S., Maio J., Anjos R., Belo J.A. (2017). Functional study of DAND5 variant in patients with Congenital Heart Disease and laterality defects. BMC Med. Genet..

[23] Kattman S.J., Witty A.D., Gagliardi M., Dubois N.C., Niapour M., Hotta A., Ellis J., Keller G. (2011). Stage-specific optimization of activin/nodal and BMP signaling promotes cardiac differentiation of mouse and human pluripotent stem cell lines. Cell Stem Cell.

[24] Lin Y., Zou J. (2020). Differentiation of Cardiomyocytes from Human Pluripotent Stem Cells in Fully Chemically Defined Conditions. STAR Protoc..

[25] Zhang Y., Cao N., Huang Y., Spencer C.I., Fu J.D., Yu C., Liu K., Nie B., Xu T., Li K. (2016). Expandable Cardiovascular Progenitor Cells Reprogrammed from Fibroblasts. Cell Stem Cell.

[26] Mansfield C., Zhao M.-T., Basu M. (2022). Translational potential of hiPSCs in predictive modeling of heart development and disease. Birth Defects Res..

[27] Cristo F., Inácio J.M., Rosas G., Carreira I.M., Melo J.B., de Almeida L.P., Mendes P., Martins D.S., Maio J., Anjos R. (2017). Generation of human iPSC line from a patient with laterality defects and associated congenital heart anomalies carrying a DAND5 missense alteration. Stem Cell Res..

[28] Inácio J.M., Almeida M., Cristo F., Belo J.A. (2020). Generation of a gene-corrected human induced pluripotent stem cell line derived from a patient with laterality defects and congenital heart anomalies with a c.455G > A alteration in DAND5. Stem Cell Res..

[29] Pars S., Cristo F., Inácio J.M., Rosas G., Carreira I.M., Melo J.B., Mendes P., Martins D.S., de Almeida L.P., Maio J. (2018). Generation and characterization of a human iPS cell line from a patient-related control to study disease mechanisms associated with DAND5 p.R152H alteration. Stem Cell Res..

[30] Lian X., Hsiao C., Wilson G., Zhu K., Hazeltine L.B., Azarin S.M., Raval K.K., Zhang J., Kamp T.J., Palecek S.P. (2012). Robust cardiomyocyte differentiation from human pluripotent stem cells via temporal modulation of canonical Wnt signaling. Proc. Natl. Acad. Sci. USA.

[31] Correia C., Koshkin A., Carido M., Espinha N., Šarić T., Lima P.A., Serra M., Alves P.M. (2016). Effective Hypothermic Storage of Human Pluripotent Stem Cell-Derived Cardiomyocytes Compatible with Global Distribution of Cells for Clinical Applications and Toxicology Testing. Stem Cells Transl. Med..

[32] Bustin S.A. (2000). Absolute quantification of mRNA using real-time reverse transcription polymerase chain reaction assays. J. Mol. Endocrinol..

[33] Minter-Dykhouse K., Nelson T.J., Folmes C.D.L. (2022). Uncoupling of Proliferative Capacity from Developmental Stage during Directed Cardiac Differentiation of Pluripotent Stem Cells. Stem Cells Dev..

[34] Fleischer S., Jahnke H.-G., Fritsche E., Girard M., Robitzki A.A. (2019). Comprehensive human stem cell differentiation in a 2D and 3D mode to cardiomyocytes for long-term cultivation and multiparametric monitoring on a multimodal microelectrode array setup. Biosens. Bioelectron..

[35] Yang X., Pabon L., Murry C.E. (2014). Engineering adolescence: Maturation of human pluripotent stem cell-derived cardiomyocytes. Circ. Res..

[36] Ahmed R.E., Anzai T., Chanthra N., Uosaki H. (2020). A Brief Review of Current Maturation Methods for Human Induced Pluripotent Stem Cells-Derived Cardiomyocytes. Front. cell Dev. Biol..

[37] Chopra A., Kutys M.L., Zhang K., Polacheck W.J., Sheng C.C., Luu R.J., Eyckmans J., Hinson J.T., Seidman J.G., Seidman C.E. (2018). Force Generation via β-Cardiac Myosin, Titin, and α-Actinin Drives Cardiac Sarcomere Assembly from Cell-Matrix Adhesions. Dev. Cell.

[38] Fenix A.M., Neininger A.C., Taneja N., Hyde K., Visetsouk M.R., Garde R.J., Liu B., Nixon B.R., Manalo A.E., Becker J.R. (2018). Muscle-specific stress fibers give rise to sarcomeres in cardiomyocytes. Elife.

[39] Weber N., Kowalski K., Holler T., Radocaj A., Fischer M., Thiemann S., de la Roche J., Schwanke K., Piep B., Peschel N. (2020). Advanced Single-Cell Mapping Reveals that in hESC Cardiomyocytes Contraction Kinetics and Action Potential Are Independent of Myosin Isoform. Stem Cell Rep..

[40] Weber N., Schwanke K., Greten S., Wendland M., Iorga B., Fischer M., Geers-Knörr C., Hegermann J., Wrede C., Fiedler J. (2016). Stiff matrix induces switch to pure β-cardiac myosin heavy chain expression in human ESC-derived cardiomyocytes. Basic Res. Cardiol..

[41] Fill M., Copello J.A. (2002). Ryanodine receptor calcium release channels. Physiol. Rev..

[42] Edokobi N., Isom L.L. (2018). Voltage-Gated Sodium Channel β1/β1B Subunits Regulate Cardiac Physiology and Pathophysiology. Front. Physiol..

[43] Kiss E., Fischer C., Sauter J.-M., Sun J., Ullrich N.D. (2022). The Structural and the Functional Aspects of Intercellular Communication in iPSC-Cardiomyocytes. Int. J. Mol. Sci..

[44] Tertoolen L.G.J., Braam S.R., van Meer B.J., Passier R., Mummery C.L. (2018). Interpretation of field potentials measured on a multi electrode array in pharmacological toxicity screening on primary and human pluripotent stem cell-derived cardiomyocytes. Biochem. Biophys. Res. Commun..

[45] Kenny B.J., Brown K.N., ECG T Wave (2022). In: StatPearls [Internet]. Treasure Island (FL): StatPearls Publishing. https://www.ncbi.nlm.nih.gov/books/NBK538264/.

[46] Mummery C., Ward-van Oostwaard D., Doevendans P., Spijker R., van den Brink S., Hassink R., van der Heyden M., Opthof T., Pera M., de la Riviere A.B. (2003). Differentiation of human embryonic stem cells to cardiomyocytes: Role of coculture with visceral endoderm-like cells. Circulation.

[47] Belo J.A., Silva A.C., Borges A.-C., Filipe M., Bento M., Gonçalves L., Vitorino M., Salgueiro A.-M., Texeira V., Tavares A.T. (2009). Generating asymmetries in the early vertebrate embryo: The role of the Cerberus-like family. Int. J. Dev. Biol..

[48] Azhar M., Schultz J.E.J., Grupp I., Dorn G.W., Meneton P., Molin D.G.M., Gittenberger-de Groot A.C., Doetschman T. (2003). Transforming growth factor beta in cardiovascular development and function. Cytokine Growth Factor Rev..

[49] Kelly R.G., Buckingham M.E., Moorman A.F. (2014). Heart fields and cardiac morphogenesis. Cold Spring Harb. Perspect. Med..

[50] Wang J., Greene S.B., Martin J.F. (2011). BMP signaling in congenital heart disease: New developments and future directions. Birth Defects Res. Part A. Clin. Mol. Teratol..

[51] Yadav M.L., Bhasker A.N., Kumar A., Mohapatra B. (2022). Identification and characterization of genetic variants of TGFB1 in patients with congenital heart disease. Meta Gene.

[52] Paige S.L., Plonowska K., Xu A., Wu S.M. (2015). Molecular regulation of cardiomyocyte differentiation. Circ. Res..

[53] Uosaki H., Cahan P., Lee D.I., Wang S., Miyamoto M., Fernandez L., Kass D.A., Kwon C. (2015). Transcriptional Landscape of Cardiomyocyte Maturation. Cell Rep..

[54] Wamstad J.A., Alexander J.M., Truty R.M., Shrikumar A., Li F., Eilertson K.E., Ding H., Wylie J.N., Pico A.R., Capra J.A. (2012). Dynamic and coordinated epigenetic regulation of developmental transitions in the cardiac lineage. Cell.

[55] Wang Y., Yi N., Hu Y., Zhou X., Jiang H., Lin Q., Chen R., Liu H., Gu Y., Tong C. (2020). Molecular Signatures and Networks of Cardiomyocyte Differentiation in Humans and Mice. Mol. Ther.-Nucleic Acids.

[56] Zhou B., Wang L. (2020). Transcriptional Profiling of Single Cardiomyocytes in Health and Disease. Curr. Cardiol. Rep..

[57] Wang L., Zhang F., Duan F., Huang R., Chen X., Ming J., Na J. (2020). Homozygous MESP1 knock-in reporter hESCs facilitated cardiovascular cell differentiation and myocardial infarction repair. Theranostics.

[58] Cai C.-L., Liang X., Shi Y., Chu P.-H., Pfaff S.L., Chen J., Evans S. (2003). Isl1 identifies a cardiac progenitor population that proliferates prior to differentiation and contributes a majority of cells to the heart. Dev. Cell.

[59] Quaranta R., Fell J., Rühle F., Rao J., Piccini I., Araúzo-Bravo M.J., Verkerk A.O., Stoll M., Greber B. (2018). Revised roles of ISL1 in a hES cell-based model of human heart chamber specification. Elife.

[60] Gao R., Liang X., Cheedipudi S., Cordero J., Jiang X., Zhang Q., Caputo L., Günther S., Kuenne C., Ren Y. (2019). Pioneering function of Isl1 in the epigenetic control of cardiomyocyte cell fate. Cell Res..

[61] Targoff K.L., Schell T., Yelon D. (2008). Nkx genes regulate heart tube extension and exert differential effects on ventricular and atrial cell number. Dev. Biol..

[62] de Sena-Tomás C., Aleman A.G., Ford C., Varshney A., Yao D., Harrington J.K., Saúde L., Ramialison M., Targoff K.L. (2022). Activation of Nkx2.5 transcriptional program is required for adult myocardial repair. Nat. Commun..

[63] Durocher D., Charron F., Warren R., Schwartz R.J., Nemer M. (1997). The cardiac transcription factors Nkx2-5 and GATA-4 are mutual cofactors. EMBO J..

[64] Rojas A., Kong S.W., Agarwal P., Gilliss B., Pu W.T., Black B.L. (2008). GATA4 is a direct transcriptional activator of cyclin D2 and Cdk4 and is required for cardiomyocyte proliferation in anterior heart field-derived myocardium. Mol. Cell. Biol..

[65] Singh M.K., Li Y., Li S., Cobb R.M., Zhou D., Lu M.M., Epstein J.A., Morrisey E.E., Gruber P.J. (2010). Gata4 and Gata5 cooperatively regulate cardiac myocyte proliferation in mice. J. Biol. Chem..

[66] Kempf H., Olmer R., Haase A., Franke A., Bolesani E., Schwanke K., Robles-Diaz D., Coffee M., Göhring G., Dräger G. (2016). Bulk cell density and Wnt/TGFbeta signalling regulate mesendodermal patterning of human pluripotent stem cells. Nat. Commun..

[67] Bedada F.B., Wheelwright M., Metzger J.M. (2016). Maturation status of sarcomere structure and function in human iPSC-derived cardiac myocytes. Biochim. Biophys. Acta.

[68] El-Battrawy I., Lan H., Cyganek L., Zhao Z., Li X., Buljubasic F., Lang S., Yücel G., Sattler K., Zimmermann W.-H. (2018). Modeling Short QT Syndrome Using Human-Induced Pluripotent Stem Cell-Derived Cardiomyocytes. J. Am. Heart Assoc..

[69] Guo F., Sun Y., Wang X., Wang H., Wang J., Gong T., Chen X., Zhang P., Su L., Fu G. (2019). Patient-Specific and Gene-Corrected Induced Pluripotent Stem Cell-Derived Cardiomyocytes Elucidate Single-Cell Phenotype of Short QT Syndrome. Circ. Res..

[70] Nakamura Y., Matsuo J., Miyamoto N., Ojima A., Ando K., Kanda Y., Sawada K., Sugiyama A., Sekino Y. (2014). Assessment of testing methods for drug-induced repolarization delay and arrhythmias in an iPS cell-derived cardiomyocyte sheet: Multi-site validation study. J. Pharmacol. Sci..

[71] Yamazaki K., Hihara T., Taniguchi T., Kohmura N., Yoshinaga T., Ito M., Sawada K. (2012). A novel method of selecting human embryonic stem cell-derived cardiomyocyte clusters for assessment of potential to influence QT interval. Toxicol. Vitr..

[72] Zhang J., Wilson G.F., Soerens A.G., Koonce C.H., Yu J., Palecek S.P., Thomson J.A., Kamp T.J. (2009). Functional cardiomyocytes derived from human induced pluripotent stem cells. Circ. Res..

[73] Yechikov S., Copaciu R., Gluck J.M., Deng W., Chiamvimonvat N., Chan J.W., Lieu D.K. (2016). Same-Single-Cell Analysis of Pacemaker-Specific Markers in Human Induced Pluripotent Stem Cell-Derived Cardiomyocyte Subtypes Classified by Electrophysiology. Stem Cells.

[74] Nijak A., Saenen J., Labro A.J., Schepers D., Loeys B.L., Alaerts M. (2021). iPSC-Cardiomyocyte Models of Brugada Syndrome-Achievements, Challenges and Future Perspectives. Int. J. Mol. Sci..

[75] Karbassi E., Fenix A., Marchiano S., Muraoka N., Nakamura K., Yang X., Murry C.E. (2020). Cardiomyocyte maturation: Advances in knowledge and implications for regenerative medicine. Nat. Rev. Cardiol..

[76] Lundy S.D., Zhu W.-Z., Regnier M., Laflamme M.A. (2013). Structural and functional maturation of cardiomyocytes derived from human pluripotent stem cells. Stem Cells Dev..

[77] Liau B., Zhang D., Bursac N. (2012). Functional cardiac tissue engineering. Regen. Med..

[78] Siedner S., Krüger M., Schroeter M., Metzler D., Roell W., Fleischmann B.K., Hescheler J., Pfitzer G., Stehle R. (2003). Developmental changes in contractility and sarcomeric proteins from the early embryonic to the adult stage in the mouse heart. J. Physiol..

[79] Yang H.-T., Tweedie D., Wang S., Guia A., Vinogradova T., Bogdanov K., Allen P.D., Stern M.D., Lakatta E.G., Boheler K.R. (2002). The ryanodine receptor modulates the spontaneous beating rate of cardiomyocytes during development. Proc. Natl. Acad. Sci. USA.

[80] Hopton C., Tijsen A.J., Maizels L., Arbel G., Gepstein A., Bates N., Brown B., Huber I., Kimber S.J., Newman W.G. (2022). Characterization of the mechanism by which a nonsense variant in RYR2 leads to disordered calcium handling. Physiol. Rep..

[81] Lee P., Klos M., Bollensdorff C., Hou L., Ewart P., Kamp T.J., Zhang J., Bizy A., Guerrero-Serna G., Kohl P. (2012). Simultaneous voltage and calcium mapping of genetically purified human induced pluripotent stem cell-derived cardiac myocyte monolayers. Circ. Res..

[82] Zhang J., Ho J.C.-Y., Chan Y.-C., Lian Q., Siu C.-W., Tse H.-F. (2014). Overexpression of myocardin induces partial transdifferentiation of human-induced pluripotent stem cell-derived mesenchymal stem cells into cardiomyocytes. Physiol. Rep..

[83] Zhang J., Klos M., Wilson G.F., Herman A.M., Lian X., Raval K.K., Barron M.R., Hou L., Soerens A.G., Yu J. (2012). Extracellular matrix promotes highly efficient cardiac differentiation of human pluripotent stem cells: The matrix sandwich method. Circ. Res..

[84] Sundnes J., Lines G.T., Grøttum P., Tveito A., Langtangen H.P., Tveito A. (2003). Electrical Activity in the Human Heart. Advanced Topics in Computational Partial Differential Equations: Numerical Methods and Diffpack Programming.

[85] Axelsen L.N., Calloe K., Holstein-Rathlou N.-H., Nielsen M.S. (2013). Managing the complexity of communication: Regulation of gap junctions by post-translational modification. Front. Pharmacol..

[86] Zhang S.-S., Shaw R.M. (2014). Trafficking highways to the intercalated disc: New insights unlocking the specificity of connexin 43 localization. Cell Commun. Adhes..

[87] Chen H.-S.V., Kim C., Mercola M. (2009). Electrophysiological challenges of cell-based myocardial repair. Circulation.

[88] Zwi L., Caspi O., Arbel G., Huber I., Gepstein A., Park I.-H., Gepstein L. (2009). Cardiomyocyte differentiation of human induced pluripotent stem cells. Circulation.

[89] Giacomelli E., Meraviglia V., Campostrini G., Cochrane A., Cao X., van Helden R.W.J., Krotenberg Garcia A., Mircea M., Kostidis S., Davis R.P. (2020). Human-iPSC-Derived Cardiac Stromal Cells Enhance Maturation in 3D Cardiac Microtissues and Reveal Non-Cardiomyocyte Contributions to Heart Disease. Cell Stem Cell.

[90] Khan M., Xu Y., Hua S., Johnson J., Belevych A., Janssen P.M.L., Gyorke S., Guan J., Angelos M.G. (2015). Correction: Evaluation of Changes in Morphology and Function of Human Induced Pluripotent Stem Cell Derived Cardiomyocytes (HiPSC-CMs) Cultured on an Aligned-Nanofiber Cardiac Patch. PLoS ONE.

[91] Hamledari H., Asghari P., Jayousi F., Aguirre A., Maaref Y., Barszczewski T., Ser T., Moore E., Wasserman W., Klein Geltink R. (2022). Using human induced pluripotent stem cell-derived cardiomyocytes to understand the mechanisms driving cardiomyocyte maturation. Front. Cardiovasc. Med..

[92] Koivisto M., Tolvanen T.A., Toimela T., Miinalainen I., Kiviaho A., Kesseli J., Nykter M., Eklund L., Heinonen T. (2022). Functional human cell-based vascularised cardiac tissue model for biomedical research and testing. Sci. Rep..

[93] Maier S.K.G., Westenbroek R.E., McCormick K.A., Curtis R., Scheuer T., Catterall W.A. (2004). Distinct subcellular localization of different sodium channel alpha and beta subunits in single ventricular myocytes from mouse heart. Circulation.

[94] Marques S., Borges A.C., Silva A.C., Freitas S., Cordenonsi M., Belo J.A. (2004). The activity of the Nodal antagonist Cerl-2 in the mouse node is required for correct L/R body axis. Genes Dev..

