# Supplementary material for: Gene-Edited Human-Induced Pluripotent Stem Cell Lines to Elucidate DAND5 Function throughout Cardiac Differentiation
Source: Cells. 2023 Feb 5;12(4):520. doi: 10.3390/cells12040520 (PMC9954670; doi:10.3390/cells12040520)
Supplement: Supplementary file 1 [file cells-12-00520-s001.zip › cells-2074844-supplementary.pdf]

Supplementary Material

Table S1 – Primer List

Gene	Sequence	Annealing Temperature (ºC)
GAPDH	Fwd 5’ G CTGGTAAAGTGGATATTGTTGCCAT 3’<br>Rv 5’ TGGAATCATATTGGAACATGTAAACC 3’	57.9
β-ACTIN	Fwd 5’ GCAAAGACCTGTACGCCAAC 3’<br>Rv 5’ AGTACTTGCGCTCAGGAGGA 3’	55
MESP1	Fwd 5’ AGCTTGGGTGCCTCCTTATT 3’<br>Rv 5’ TGCTTCCCTGAAAGACATCA 3’	63.1
ISL1	Fwd 5’ AGATTATATCAGGTTCTACGGGATCA 3’<br>Rv 5’ ACACAGCGGAAACACTCGAT 3’	57.9
NKX2.5	Fwd 5’ CAAGTGTGCGTCTGCCTTT 3’<br>Rv 5’ CAGCTCTTTCTTTTCGGCTCTA 3’	64.3
GATA4	Fwd 5’ TAGACCGTGGGTTTTGCATTG 3’<br>Rv 5’ CATCCAGGTACATGGCAAACAG 3’	57.9
TNNT2	Fwd 5’ TTCACCAAAGATCTGCTCCTCGCT 3’<br>Rv 5’ TTATTACTGGTGTGGAGTGGGTGTGG 3’	57.9
MYH6	Fwd 5’ GTCATTGCTGAAACCGAGA 3’<br>Rv 5’ GCTCCTTGAGGTTGAAAAGCA 3’	57.9
MYH7	Fwd 5’ ACATCATCACCCATGGAGACGAGA 3’<br>Rv 5’ GCAACAGAGTTTATTGAAAAGCA 3’	57.9
TNNI1	Fwd 5’ ATGGAATAGGAGGACAGTAGGT 3’<br>Rv 5’ AAGAATCCAGGGCAGTAAGC 3’	59.9
TNNI3	Fwd 5’ CTGCAGATTGCAAAGCAAGA 3’<br>Rv 5’ CCTCCTTCTTCACCTGCTTG 3’	59
SCN1B	Fwd 5’ GACCAACGCTGAGACCTTCA 3’<br>Rv 5’ TCCAGCTGCAACACCTCATT 3’	67.5
RYR2	Fwd 5’ GTTGTCATGATGAGGAAGATGACG 3’<br>Rv 5’ CTTTGCTGGCACTGATTGTCTG 3’	67.2
DAND5	Fwd 5’ GTCGACTGCTAGTGACCTTGAG 3’<br>Rv 5’ TCAGGTGGAGGATACAGGACTT 3’	60
gRNA-DAND5	AAACGCTTAGCGGGGCCCTGCCTAC	-
ssODN (template)	ATGCTCCTTGGCCAGCTATCCACTCTTCTGTGCCTGCTTGCTAATAATAACCTACAGGATCCGG-GAGGCCTGAACCCCAGTCTCCTCGACCTCAG-TCCTGGGCTGCAG	-

Table S2 – Antibody List

	Antibody	Host	Dilu-tion	Company Cat # and RRID
Primary Antibodies	NANOG	Rabbit	1:50	Abcam Cat# ab21624, RRID:AB_446437
	OCT4	Rabbit	1:400	Abcam Cat# ab19857, RRID:AB_445175
	SSEA4	Mouse	1:200	Abcam Cat# ab16287, RRID:AB_778073
	SMA	Mouse	1:600	Dako Cat#M0851, RRID:AB_2223500
	AFP	Rabbit	1:200	Dako Cat#A0008, RRID:AB_2650473
	TUBB3	Mouse	1:100	Sigma-Aldrich Cat# 065M4820V
	α-ACTININ	Mouse	1:800	Sigma-Aldrich Cat# A7811, RRID:AB_476766
	MLC2v	Rabbit	1:200	Proteintech Cat# 10906-1-AP, RRID:AB_2147453
	TNNT2	Mouse	1:250	Thermo Fisher Scientific Cat# MA5-12960, RRID:AB_11000742
	Cx43	Goat	1:500	SICGEN Cat# AB0016
Secondary Antibodies	Alexa Fluor 488-conjugated Donkey anti-mouse IgG (H + L)	Donkey	1:300	Jackson ImmunoResearch Labs Cat# 715-545-150, RRID:AB_2340846
	Alexa Fluor 488-conjugated Donkey anti-Rabbit IgG (H + L)	Donkey	1:300	Jackson ImmunoResearch Labs Cat# 711-545-152, RRID:AB_2313584
	Alexa Fluor 594-conjugated Donkey anti-Mouse IgG (H + L)	Donkey	1:300	Thermo Fisher Scientific Cat# A-21203, RRID:AB_141633
	Alexa Fluor 633-conjugated Donkey anti-Goat IgG (H + L)	Donkey	1:300	Thermo Fisher Scientific Cat# A21082, RRID:AB_10562400
